# Carpal Tunnel Syndrome as a Potential Indicator of Cardiac Amyloidosis: A Systematic Review and Meta-Analysis

**DOI:** 10.7759/cureus.75582

**Published:** 2024-12-12

**Authors:** Mohamed A Elghouneimy, Nagham Bushara, Omar A Abdelwahab, Ayman A Makableh, Doaa M Alnabwy, Rehab A Diab

**Affiliations:** 1 Plastic Surgery, Cambridge University, London, GBR; 2 Medicine, Zagazig University, Zagazig, EGY; 3 Internal Medicine, Al Azhar University, Cairo, EGY; 4 Nursing, Dibba Hospital, Al Fujairah, ARE; 5 Cardiology, Al Azhar University, Cairo, EGY

**Keywords:** amyloidosis, cardiac amyloidosis, carpal tunnel syndrome, meta-analysis, neuropathy

## Abstract

Carpal tunnel syndrome (CTS) and cardiac amyloidosis (CA) are seemingly disparate medical conditions but may be linked. CTS can be a sign of early CA, and CA can be a hidden cause of heart failure. Therefore, in this systematic review and meta-analysis, we aim to investigate the expected correlation between the occurrence of CTS and CA.

A comprehensive search was conducted across multiple databases, including PubMed, Web of Science, Scopus, and Cochrane Library, to get relevant studies previously published before June 2023. No language restrictions were applied. Randomized clinical trials and observational studies have been included to investigate the proportion of patients reporting CA among patients with an established diagnosis of CA, and also the incidence of patients who have CTS among patients with CA has been investigated with a pooled estimation of the expected time from the diagnosis of CTS till the development of CA. Studies that did not report data on CTS or CA or lacked sufficient details were excluded. Meta-analysis of data for each outcome was performed using R version R.4.3.2 software (R Foundation for Statistical Computing, Vienna, Austria) using the Meta package. Heterogeneity across studies was assessed using the I² statistic.

A meta-analysis of 15 studies, including 1416 patients, evaluated the relationship between CTS and CA. Among these, 495 patients with CTS were assessed for the presence of CA, and 915 patients with CA were evaluated for the presence of CTS. The pooled meta-analysis of eight studies, which included 915 patients with CA, revealed that 38% (95% CI: 35%-41%) had a history of CTS. Conversely, the proportion of patients with CTS who developed CA was 13% (95% CI: 4%-35%). The pooled mean time from CTS to the development of CA, based on 639 patients across four studies, was 6.02 years (95% CI: 3.76-8.36). Significant heterogeneity was noted for some outcomes (e.g., proportion of CA in patients with CTS: I²=93%), likely influenced by variations in study populations, age distributions, and diagnostic criteria.

Our review of the literature suggests that there may be a link between CTS and CA. However, more research is needed to confirm this link and to understand how the two conditions are related. It is important to consider the possibility of CA in patients with CTS, as screening, early detection, and timely treatment can improve outcomes by slowing disease progression and reducing complications.

## Introduction and background

In recent years, the intersection of clinical observations and investigative research has fostered an intriguing nexus between seemingly disparate medical conditions. Carpal tunnel syndrome (CTS), a common peripheral nerve disorder, has been recognized for a long time as a clinical entity predominantly related to mechanical compression of the median nerve at the wrist. It significantly impacts the quality of life by causing pain, numbness, and functional impairments that hinder daily activities [1,2]. In contrast, cardiac amyloidosis (CA) is a group of systemic disorders characterized by the extracellular deposition of abnormal protein fibrils in cardiac tissues. It contributes to heart failure through infiltrative cardiomyopathy, conduction abnormalities, and diastolic dysfunction, often resulting in delayed diagnosis and poor outcomes [3,4]. It has attracted considerable attention due to its insidious progression and frequently delayed diagnosis [4,5].

CA is considered the most pernicious late manifestation of systemic amyloidosis, particularly wild-type transthyretin amyloidosis (ATTRwt) [5]. Amyloid fibril deposition in the cardiac tissue results in a progressive and infiltrative form of cardiomyopathy characterized by increased ventricular wall thickness, diastolic dysfunction, and cardiac conduction system disorders [6-8]. Recent studies identified CA as an underrecognized cause of heart failure [9-11]. The deposition of amyloid fibrils in connective tissues, including the transverse carpal ligament, may contribute to the mechanical compression of the median nerve observed in CTS. This localized amyloid accumulation in peripheral tissues often precedes the systemic involvement seen in CA, including cardiac tissue infiltration, by several years. This temporal relationship suggests that CTS could serve as an early clinical marker for CA [12].

Additionally, 88% of patients with ATTRwt cardiomyopathy who underwent regular neuropathy screening at the time of their ATTRwt diagnosis (neurologist assessment and nerve conduction testing) were found to have CTS (36 of 41), compared to 7% of age-matched controls [12]. On the other hand, the prevalence of ATTRwt cardiomyopathy in men with bilateral CTS was 4%, rising to 33% in those with left ventricular hypertrophy. This suggests that amyloid fibril deposition in hand tissues precedes deposition in cardiac tissues for a considerable period [13]. Consequently, CTS manifestations often appear 5-10 years before the symptoms of CA, making CTS a potentially valuable tool for early screening and detection of CA [14-18].

Anticipation and early recognition of CA are very valuable in developing a therapeutic plan with proven efficacy and saving more time for ATTRwt amyloidosis patients [18]. Thus, the aim of this systematic review and meta-analysis study is to confirm the relationship between CTS and CA and draw the attention of the medical society toward the possibility of CTS being a prediction tool for CA.

## Review

Methods

We followed the Preferred Reporting Items for Systematic Reviews and Meta-Analysis (PRISMA) statement guidelines [19]. During this systematic review and meta-analysis, the methods were done in strict accordance with the Cochrane Handbook of Systematic Reviews and Meta-analysis [20]. All steps of this study were specified, and the protocol was registered on PROSPERO (ID: CRD42023456840).

Eligibility Criteria

We included all studies that fulfilled the following criteria:

Population: Studies with patients who had CTS or CA.

Observation: Studies were included if patients developed CA in the presence of CTS or if they developed CTS in the presence of CA. For CA, confirmation methods included echocardiography, cardiac magnetic resonance imaging (MRI), endomyocardial biopsy, or technetium-labeled bone scintigraphy. For CTS, diagnoses were confirmed via clinical examination, nerve conduction studies, or electromyography.

Outcome: Studies that reported the relationship between CA and CTS.

Study design: Observational studies that focus on the relationship between CA and CTS.

We excluded studies reported as abstracts only, review articles, letters to the editor, case reports, case series, and studies not published in English.

Literature Search

We conducted our study using four electronic databases: PubMed, Web of Science, Scopus, and Cochrane Library from inception until 1 June 2023 using the following search strategy: ((Carpal Tunnel Syndrome) OR (Median Neuropathy) OR (Median nerve compression) OR (Carpal tunnel entrapment) OR (Carpal tunnel neuropathy) OR (Entrapment Neuropathy) OR (Compression Neuropathy) OR (tenosynovitis)) AND ((cardiac amyloidosis) OR (amyloid cardiomyopathy) OR (amyloidosis) OR (transthyretin) OR (light chain) OR (ATTR) OR (AL)). Additionally, we screened the references of the included studies for any possible eligible studies.

Study Selection

After removing duplicates using EndNote software (Clarivate, Philadelphia, PA), all search results were assessed for eligibility through title/abstract screening followed by full-text screening to ensure data reliability for analysis. Each screening was conducted by more than one reviewer using the Rayyan website in a blinded manner. Additionally, manual screening of references of included studies was independently performed by two reviewers to identify additional eligible studies. Any discrepancies were resolved through discussion or consultation with a third reviewer.

Data Collection Process and Data Items

A data extraction sheet was constructed on Excel software (Microsoft, Redmond, WA, USA). The data extraction includes the following domains: (1) study ID, (2) study year, (3) country, (4) study design, (5) sample size, (6) age, (7) BMI, (8) type of intervention, (9) baseline labs, (10) echocardiographic assessment, (11) cardiomyopathy, and (12) CTS.

Risk of Bias Assessment

NIH tool of quality assessment for single-arm observational studies is recommended for assessing the quality of observational studies in meta-analysis [21]. The NIH tool for single-arm observational studies is a 14-question tool that assesses the quality of the study design and methods. It covers a wide range of areas, including the clarity of the study question and population definition, participation rate, sampling and recruitment methods, sample size justification, exposure and outcome measurement, timeframe, dose-response relationship, exposure and outcome definition, validity, reliability, and consistency, repeated exposure measurement, outcome assessor blinding, loss to follow-up, and confounding variable control. Two reviewers independently assessed the risk of bias for each included study. Any disagreements were resolved through discussion and consensus. In cases where consensus could not be reached, a third independent reviewer was consulted to make the final determination.

Statistical Analysis

The DerSimonian-Laird meta-analysis approach was used to calculate the pooled effect size for each outcome in our study [22]. For dichotomous outcomes, odds ratios (ORs) with 95% CI were calculated and pooled. For continuous outcomes, means with 95% CI were pooled using this approach. This model was chosen because it allows for a larger standard error in the pooled estimate, which accounts for any variability and dispute in the estimations. The DerSimonian-Laird random-effects model was chosen over a fixed-effects model because it accounts for both within-study and between-study variability. This approach is suitable when there is significant heterogeneity among studies, as it assumes that the true effect size may vary from one study to another due to differences in study populations, interventions, and methodologies. We performed the meta-analysis, created funnel plots, and evaluated publication bias using R Studio version 4.2.3 and the Meta Package's "metaprop" and "metamean" functions.

Statistical heterogeneity among the included studies was assessed using the Chi-square test (Cochrane Q test). The I-squared value was then calculated using the Cochrane Q statistic and the following formula: I^2^=(Q-df)/Qx100%. Significant heterogeneity was defined as a Chi-square P-value of less than 0.1, while high heterogeneity was defined as I-squared values more than or equal to 50%. Publication bias assessment was non-applicable as the number of included studies for each outcome was less than 10 studies [23,24].

Results

Literature Search Results

**Figure 1:**
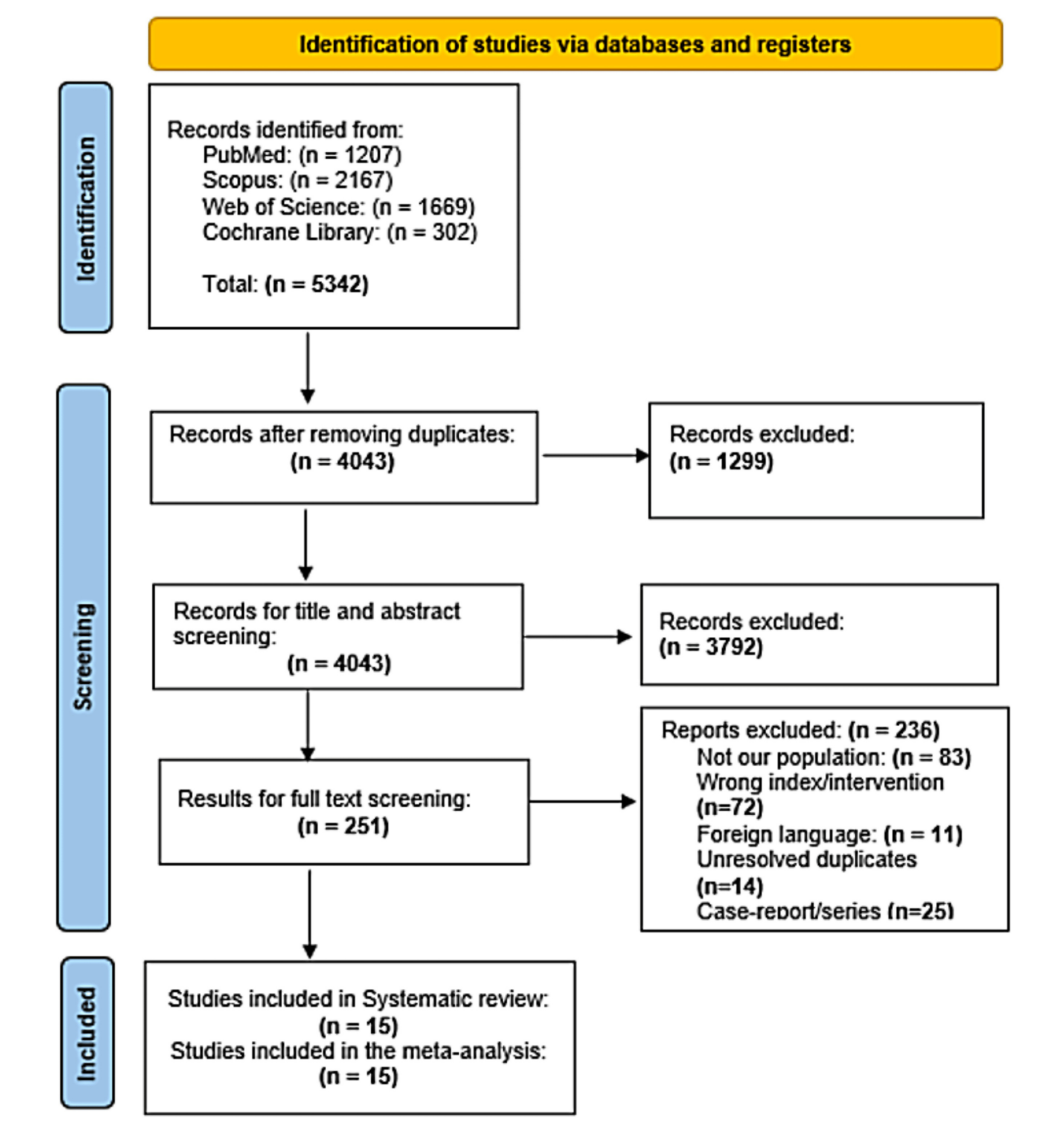
PRISMA flow diagram PRISMA: Preferred Reporting Items for Systematic Reviews and Meta-Analysis

Our literature search revealed 4043 results after duplicate removal. Upon title and abstract review, 251 papers were selected for full-text review. The final number of included studies in systematic review and meta-analysis comprised 15 of these investigations. No further papers were included after manually searching the references of the included studies. Figure 1 depicts the PRISMA flow diagram of the study selection process.

Characteristics of the Included Studies

Fifteen studies were included in the meta-analysis, comprising a total of 1416 patients: 495 with CTS who were evaluated for the presence of CA and 915 with CA who were evaluated for the presence of CTS. A summary of the characteristics of the included studies is provided in Table 1 and Table 2. Overall, the quality of the included studies ranked as Good Quality according to the NIH quality assessment tool of observational studies (Table 3 of Appendices).

**Table 1:** Summary table of included studies ATTR-CM: transthyretin amyloid cardiomyopathy; CTS: carpal tunnel syndrome; ATTRwt: wild-type transthyretin amyloidosis; mTTR-FAC: mutated transthyretin familial amyloid cardiomyopathy; TTR-CA: transthyretin cardiac amyloidosis; AS: aortic stenosis; AL: light chain amyloidosis

Study ID	Study design	Country	Sample size	Population	Type of mutation	Main findings
Benedicto, 2022 [25]	Retrospective cohort	Spain	300	ATTR-CM patients	Hereditary ATTR-CM and wild-type ATTR-CM	38% of the cohort had CTS
Bishop, 2018 [26]	Retrospective cohort	USA	82	Patients with biopsies proved CA	ATTR and AL	40.2% of the cohort had CTS
Campagnolo, 2023 [27]	Cross-sectional	Italy	16	Patients with ATTRwt, with no risk factors for neuropathy	Wild-type ATTR-CM	81.25% of the cohort had CTS
Damy, 2015 [28]	Cross-sectional	France	17	Patients with mTTR-FAC	Amyloidogenic mTTR	35.3% of the cohort had CTS
Galat, 2016 [29]	Retrospective cohort	France	16	Patients with AS and TTR-CA	13 with WT-TTR, one Val122I mutation, and three did not have genetic sequencing of TTR	31% of the cohort had CTS
Ladefoged, 2023 [30]	Case-control	Denmark	120	Patients scheduled for surgery for idiopathic CTS	ATTRwt	8.33% of the cohort had ATTRwt
Reyes, 2017 [31]	Prospective cohort	USA	58	Patients undergoing carpal tunnel decompression	ATTRwt and AL	3.4% of the cohort had CA
Shije, 2022 [32]	Cross-sectional	USA	16	Patients with bilateral CTS	V122I	12.5% of the cohort had CA
Siepen, 2019 [14]	Cross-sectional	Germany	389	Patients with mutant-ATTR and wild-type ATTR	p.Val50Met, p.Val40Ile, and p.Leu78His	36.2% of the cohort had CTS
Sperry, 2018 [33]	Prospective cohort	USA	98	Patients undergoing carpal tunnel release	ATTR and AL	10.2% of the cohort had CA
Sperry, 2021 [34]	Prospective cohort	USA	13	Patients undergoing carpal tunnel release	ATTR	15.4% of the cohort had ATTR
Vianello, 2021 [13]	Retrospective cohort	Italy	53	Male patients underwent bilateral carpal tunnel surgery	ATTR	3.8% of the cohort had ATTRwt
Wang, 2022 [35]	Retrospective cohort	China	29	Patients with hereditary ATTR-CM	Ala97Ser	20.7% of the cohort had CTS
Zadok, 2020 [36]	Case-control	Israel	108	Patients with systemic amyloidosis (AL or ATTR)	ATTR and AL	42.4% of the CA cohort had CTS and 78% of the CTS cohort had CA
Zegri-Reiriz, 2019 [37]	Cross-sectional	Spain	101	Patients who had undergone CTS surgery	ATTR and AL	3% of the cohort had CA

**Table 2:** Baseline table of included studies in the systematic review and meta-analysis

Study ID	Sample size	Age (years), mean (SD)	Sex (male), n (%)	History of DM, n (%)	History of HTN, n (%)	History of AF or flutter, n (%)	History of CAD, n (%)	Time from CTS till CA diagnosis (years), mean (SD)
Benedicto, 2022 [25]	300	Median (IQR): 78 (72-84)	253 (84.3)	NR	196 (65.3)	188 (62.7)	47 (15.7)	NR
Bishop, 2018 [26]	82	70.6 (9.8)	55 (67)	14 (17.1)	55 (67.1)	Cardiac comorbidity: 40 (48.8)		NR
Campagnolo, 2023 [27]	16	Median (range): 73.5 (65-86)	16 (100)	NR	NR	NR	NR	NR
Damy, 2015 [28]	17	73 (6.9)	13 (76.5)	NR	10 (85.8)	NR	NR	NR
Galat, 2016 [29]	16	79 (6)	13 (81)	NR	NR	9 (56)	NR	NR
Ladefoged, 2023 [30]	120	74.5 (6.1)	109 (90.8)	15 (12.5)	65 (54.2)	11 (9.2)	18 (15.0)	4.2 (1.3)
Reyes, 2017 [31]	58	NR	NR	NR	NR	NR	NR	NR
Shije, 2022 [32]	16	Mean (range): 55.75 (38-73).	2 (12.5)	3 (18.75)	NR	NR	10 (62.5)	NR
Siepen, 2019 [14]	389	68.8 (11.2)	356 (91.5)	NR	NR	NR	NR	7 (10.3)
Sperry, 2018 [33]	98	Median (IQR): 68 (61-74)	51 (52)	27 (28)	69 (70)	12 (12)	18 (18)	NR
Sperry, 2021 [34]	13	65.5 (8.1)	NR	NR	NR	NR	NR	NR
Vianello, 2021 [13]	53	72.0 (12.2)	53 (100)	6 (11)	30 (57)	7 (13)	6 (11)	4.4 (2.3)
Wang, 2022 [35]	29	Median (IQR): 56 (47.8-66.3)	27 (93.1)	NR	NR	2 (7)	NR	4.6 (1.9)
Zadok, 2020 [36]	108	Median (IQR): 68 (61, 78)	68 (63)	NR	NR	NR	NR	4.62 (3)
Zegri-Reiriz, 2019 [37]	101	Median (IQR): 69 (64-77)	32 (31.7)	32 (31.7)	65 (64.4)	7 (6.9)	NR	11.5 (2.33)

Outcomes

**Figure 2:**
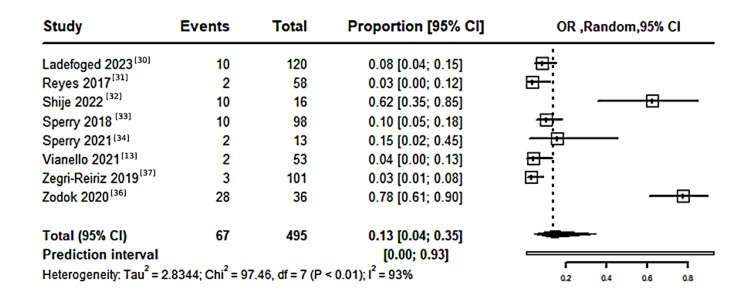
Forest plot illustrating the proportion of CA in patients with CTS CTS: carpal tunnel syndrome; CA: cardiac amyloidosis

CA in patients with CTS: A pooled meta-analysis of eight studies, including 495 patients with CTS, showed that the proportion of patients developing CA was 13% (95% CI: 4%-35%), with significant heterogeneity among studies (I²=93%, P<0.01), as shown in Figure 2.

**Figure 3:**
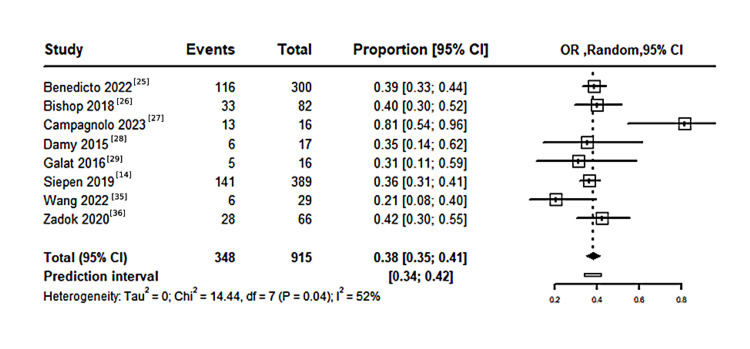
Forest plot illustrating the proportion of history of CTS in population with CA CTS: carpal tunnel syndrome; CA: cardiac amyloidosis

**Figure 4:**
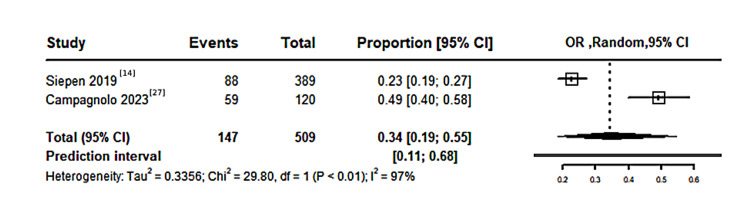
Forest Plot illustrating the proportion of history of CTS in the population with CA CTS: carpal tunnel syndrome; CA: cardiac amyloidosis

History of CTS in patients with CA: A pooled meta-analysis of 915 patients with CA, included in eight studies, revealed that the proportion of patients with a history of CTS was 38% (95% CI: 35%-41%), with statistically significant moderate heterogeneity (I²=52%, P=0.04), as shown in Figure 3. Meanwhile, a pooled meta-analysis of 509 patients with CA, based on two studies reporting a history of bilateral CTS, found the proportion with a history of bilateral CTS to be 34% (95% CI: 19%-55%), with significant heterogeneity (I²=97%, P<0.01), as shown in Figure 4.

**Figure 5:**
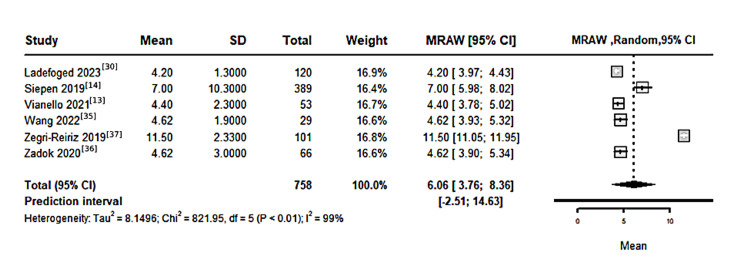
Forest plot for the time from CTS till the development of CA CTS: carpal tunnel syndrome; CA: cardiac amyloidosis

Time from CTS to development of CA: The pooled mean time from CTS to the development of CA, based on 639 patients across four studies, was 6.02 years (95% CI: 3.76-8.36), with significant heterogeneity among studies (I²=99%, P<0.01), as shown in Figure 5.

Discussion

Significance of the Study

To the best of our knowledge, this is the first systematic review and meta-analysis that evaluates the presence of CTS in patients with CA and the presence of CA in patients with CTS. Although this study could not assess the relationship between the two findings or evaluate the predictive power of CTS in detecting CA, this study opens the door for future research to evaluate this.

Summary of Findings

We found that approximately 13% of patients with CTS developed CA, indicating a potential association between the conditions. The mean time from CTS diagnosis to the development of CA was approximately 7.02 years. Additionally, around 38% of patients with CA had a history of CTS.

Explanation of the Finding and Implications of These Findings in Practice

Systemic amyloidosis is a group of rare and complex diseases characterized by the anomalous accumulation of amyloid proteins throughout the body's organs and tissues. There are multiple organs involved in this disease including the kidney, heart, gastrointestinal system, neurological system, liver, spleen, soft tissue, and skin. In the case of CTS, amyloid deposits in the transverse carpal ligament and surrounding soft tissue may contribute to mechanical compression of the median nerve, while in CA, the deposition of amyloid proteins within the myocardium results in progressive cardiac dysfunction [2,5-8]. This shared mechanism suggests that CTS could serve as an early manifestation of systemic amyloidosis, reflecting early peripheral tissue involvement that may precede cardiac amyloid infiltration by several years. Our findings suggest a potential temporal relationship between CTS and the development of CA in those patients. In other words, is CTS could be an indicator of the involvement of cardiac muscle in patients with systemic amyloidosis? Could the presence of CTS be a mirror for the presence of CA? The observed proportion of CA development among CTS patients, approximately 13%, is noteworthy. While this proportion may not indicate a direct causal link, it raises intriguing questions about the shared mechanisms or risk factors that could underlie both conditions. On another hand, 38% of patients with CA had a history of CTS, with a mean time of seven years from CTS diagnosis to the development of CA. However, it is essential to note that our findings do not establish causation, and further research is required to elucidate the precise mechanisms involved.

The clinical ramifications of our results are significant. If CTS is indeed associated with CA, clinicians should consider performing a more comprehensive assessment of patients presenting with CTS, particularly those with risk factors for CA, such as age or family history. Early detection of CA in patients with CTS could result in earlier interventions, which may enhance patient outcomes and quality of life. Emerging diagnostic methods for CTS, such as nerve conduction studies and high-resolution ultrasound imaging, offer opportunities to identify CTS cases early. Integrating these diagnostic tools with screening for amyloid deposition could enhance early detection of CA in at-risk patients, particularly those with bilateral CTS or unexplained neuropathic symptoms. In addition, these results emphasize the significance of an interdisciplinary approach to patient care. It may be necessary for cardiologists, neurologists, and orthopedic specialists to collaborate in order to provide comprehensive care to patients with both CTS and CA. In addition, educating patients about the possible link between these conditions can empower them to seek prompt medical care and monitoring. 

Significant heterogeneity was observed in our meta-analysis. The sources of heterogeneity may be attributed to several factors: significant heterogeneity was observed in our meta-analysis, particularly in the proportion of CTS patients who developed CA. The sources of this heterogeneity may be attributed to several factors. First, differences in study designs played a role, as the included studies varied in type, which may contribute to methodological variability. Second, variations in diagnostic criteria, such as differences in how CTS and CA were diagnosed across studies (e.g., clinical assessment, nerve conduction studies, imaging techniques, and biopsy confirmation), could introduce additional heterogeneity. Furthermore, patient characteristics, including variations in demographics such as age ranges, sex distribution, genetic mutations (e.g., ATTRwt vs. ATTRm), and the presence of comorbidities like diabetes mellitus or hypertension, were another source of variability. Additionally, the included studies had varying sample sizes, which can affect the weight of each study in the meta-analysis and contribute to heterogeneity.

Strength Points and Limitations

This study is the first meta-analysis investigating the probability of occurrence of CA in patients diagnosed with CTS and time interval to the first diagnosis and the possible association between two events. One of the most important limitations of this study is the variability among the included studies, including differences in study design and patient populations, which may contribute to significant heterogeneity, introduce bias, and limit the generalizability of our findings. Moreover, the limited number of studies for certain outcomes and the retrospective nature of the included studies may compromise the validity of our conclusions. Additionally, we did not include unpublished studies, which may introduce bias. 

Recommendations for Future Research and Clinical Practice

Future studies could employ prospective cohort designs to establish the temporal and the causal link between CTS and CA, investigating potential mechanisms, and identifying specific risk factors. Longitudinal investigations that track the development of CA in CTS patients over an extended period of time can provide stronger evidence. Future analyses could stratify studies by diagnostic criteria, patient demographics, and geographical location to better understand the sources of heterogeneity.

To provide comprehensive care for patients with both CTS and CA, collaboration between multidisciplinary teams of clinicians, including cardiologists, neurologists, and orthopedic specialists, should be encouraged. Specific areas of collaboration could include the development of joint guidelines for evaluating CTS in patients with systemic amyloidosis and incorporating screening protocols for CA in patients presenting with bilateral or atypical CTS. Regular multidisciplinary case discussions and shared decision-making frameworks can enhance diagnostic accuracy and streamline care pathways. It is crucial to establish standard diagnostic criteria for CTS and CA in order to facilitate accurate comparisons between studies. Potential biomarkers for early detection of CA in CTS patients include serum and urine-free light chains for AL amyloidosis and transthyretin (TTR) levels for ATTR amyloidosis. Genetic testing for TTR mutations may also be beneficial in identifying hereditary forms of ATTR. Imaging modalities such as cardiac MRI with late gadolinium enhancement and technetium-99m bone scintigraphy can detect early cardiac involvement in systemic amyloidosis. High-resolution ultrasound of the wrist may identify amyloid deposits in CTS patients, providing a non-invasive tool to support early diagnosis of systemic amyloidosis.

## Conclusions

Our systematic review and meta-analysis suggest a potential association between CTS and CA. Although these findings are encouraging, they do not establish a causal relationship. They highlight the significance of contemplating CA risk in patients with CTS, as well as the potential benefits of early detection and intervention. To elucidate the relationship between these conditions and the mechanisms involved, additional research is required. Our study contributes to the growing corpus of evidence in this field and emphasizes the need for additional research.

## Appendices

**Table 3:** Quality assessment of the included studies NIH tool for single-arm observational studies Q1: Was the study question or objective clearly stated? Q2: Was the study population clearly specified and defined? Q3: Was the participation rate of eligible persons at least 50%? Q4: Were all the subjects selected or recruited from the same or similar populations (including the same time period)? Were inclusion and exclusion criteria for being in the study prespecified and applied uniformly to all participants? Q5: Was a sample size justification, power description, or variance and effect estimates provided? Q6: For the analyses in this paper, was the exposure(s) of interest measured prior to the outcome(s) being measured? Q7: Was the timeframe sufficient so that one could reasonably expect to see an association between exposure and outcome if it existed? Q8: For exposures that can vary in amount or level, did the study examine different levels of the exposure as related to the outcome (e.g., categories of exposure or exposure measured as a continuous variable)? Q9: Were the exposure measures (independent variables) clearly defined, valid, reliable, and implemented consistently across all study participants? Q10: Was the exposure(s) assessed more than once over time? Q11: Were the outcome measures (dependent variables) clearly defined, valid, reliable, and implemented consistently across all study participants? Q12: Were the outcome assessors blinded to the exposure status of participants? Q13: Was loss to follow-up after baseline 20% or less? Q14: Were key potential confounding variables measured and adjusted statistically for their impact on the relationship between exposure(s) and outcome(s)? Y, yes; N, no; NA, not applicable; CD: cannot be determined

Study ID	Quality assessment for single-arm observational studies according to the NIH tool															
	Q1	Q2	Q3	Q4	Q5	Q6	Q7	Q8	Q9	Q10	Q11	Q12	Q13	Q14	Overall score	Overall quality
Benedicto, 2022 [25]	Y	Y	Y	Y	Y	CD	Y	NA	Y	N	Y	N	CD	N	8	Good
Bishop, 2018 [26]	Y	Y	Y	Y	Y	Y	Y	NA	Y	Y	Y	Y	Y	Y	13	Good
Campagnolo, 2023 [27]	Y	Y	Y	Y	CD	Y	Y	NA	Y	N	Y	N	Y	Y	10	Good
Damy, 2015 [28]	Y	Y	Y	Y	Y	N	CD	NA	Y	N	Y	N	CD	Y	8	Good
Galat, 2016 [29]	Y	Y	Y	Y	CD	Y	Y	NA	Y	N	Y	CD	Y	N	9	Good
Ladefoged, 2023 [30]	Y	Y	Y	Y	Y	Y	Y	NA	Y	N	Y	N	Y	CD	10	Good
Reyes, 2017 [31]	Y	Y	Y	Y	Y	Y	Y	NA	Y	N	Y	N	Y	CD	10	Good
Shije, 2022 [32]	Y	Y	Y	Y	CD	Y	Y	NA	Y	N	Y	N	Y	CD	9	Good
Siepen, 2019 [14]	Y	Y	Y	Y	Y	Y	Y	NA	Y	N	Y	CD	Y	CD	10	Good
Sperry, 2018 [33]	Y	Y	Y	Y	Y	N	CD	NA	Y	N	Y	Y	CD	CD	8	Good
Sperry, 2021 [34]	Y	Y	Y	Y	Y	N	CD	NA	Y	N	Y	N	Y	CD	8	Good
Vianello, 2021 [13]	Y	Y	Y	Y	Y	Y	Y	NA	Y	N	Y	N	Y	CD	10	Good
Wang, 2022 [35]	Y	Y	Y	Y	Y	Y	Y	NA	Y	N	Y	CD	Y	CD	10	Good
Zadok, 2020 [36]	Y	Y	Y	Y	Y	Y	Y	NA	Y	N	Y	Y	Y	N	11	Good
Zegri-Reiriz, 2019 [37]	Y	Y	Y	Y	Y	Y	Y	NA	Y	CD	Y	N	Y	Y	11	Good

## References

[1] Sevy JO, Sina RE, Varacallo M (2024). Carpal Tunnel Syndrome. https://pubmed.ncbi.nlm.nih.gov/28846321/#:~:text=Carpal%20tunnel%20syndrome%20(CTS)%20occurs%20when.

[2] Genova A, Dix O, Saefan A, Thakur M, Hassan A (2020). Carpal tunnel syndrome: a review of literature. Cureus.

[3] Shams P, Ahmed I (2024). Cardiac Amyloidosis. https://pubmed.ncbi.nlm.nih.gov/35593829/#:~:text=Cardiac%20amyloidosis%20is%20the%20most%20common.

[4] Siddiqi OK, Ruberg FL (2018). Cardiac amyloidosis: an update on pathophysiology, diagnosis, and treatment. Trends Cardiovasc Med.

[5] Nienhuis HL, Bijzet J, Hazenberg BP (2016). The prevalence and management of systemic amyloidosis in Western countries. Kidney Dis (Basel).

[6] Jain A, Zahra F (2023). Transthyretin Amyloid Cardiomyopathy (ATTR-CM). https://www.ncbi.nlm.nih.gov/books/NBK574531/#:~:text=Transthyretin%20amyloid%20cardiomyopathy%20is%20a%20rare.

[7] Bejar D, Colombo PC, Latif F, Yuzefpolskaya M (2015). Infiltrative cardiomyopathies. Clin Med Insights Cardiol.

[8] Fikrle M, Paleček T, Kuchynka P, Němeček E, Bauerová L, Straub J, Ryšavá R (2013). Cardiac amyloidosis: a comprehensive review. Cor Vasa.

[9] Porcari A, Bussani R, Merlo M, Varrà GG, Pagura L, Rozze D, Sinagra G (2021). Incidence and characterization of concealed cardiac amyloidosis among unselected elderly patients undergoing post-mortem examination. Front Cardiovasc Med.

[10] Raval M, Siddiq S, Sharma K (2023). A review of recent advances in the diagnosis of cardiac amyloidosis, treatment of its cardiac complications, and disease-modifying therapies. F1000Res.

[11] González-López E, Gallego-Delgado M, Guzzo-Merello G (2015). Wild-type transthyretin amyloidosis as a cause of heart failure with preserved ejection fraction. Eur Heart J.

[12] Russell A, Hahn C, Chhibber S, Korngut L, Fine NM (2021). Utility of neuropathy screening for wild-type transthyretin amyloidosis patients. Can J Neurol Sci.

[13] Vianello PF, La Malfa G, Tini G (2021). Prevalence of transthyretin amyloid cardiomyopathy in male patients who underwent bilateral carpal tunnel surgery: the ACTUAL study. Int J Cardiol.

[14] Aus dem Siepen F, Hein S, Prestel S (2019). Carpal tunnel syndrome and spinal canal stenosis: harbingers of transthyretin amyloid cardiomyopathy?. Clin Res Cardiol.

[15] Cappelli F, Zampieri M, Fumagalli C (2021). Tenosynovial complications identify TTR cardiac amyloidosis among patients with hypertrophic cardiomyopathy phenotype. J Intern Med.

[16] Westin O, Fosbøl EL, Maurer MS (2022). Screening for cardiac amyloidosis 5 to 15 years after surgery for bilateral carpal tunnel syndrome. J Am Coll Cardiol.

[17] Gannon NP, Ward CM (2024). Results of implementation of amyloidosis screening for patients undergoing carpal tunnel release. J Hand Surg Am.

[18] Kuznecova I, Mierkyte G, Janciauskas D (2023). Impact of carpal tunnel syndrome surgery on early diagnosis and treatment of transthyretin cardiac amyloidosis. Medicina (Kaunas).

[19] Page MJ, McKenzie JE, Bossuyt PM (2021). The PRISMA 2020 statement: an updated guideline for reporting systematic reviews. BMJ.

[20] Cheng J, Cai M, Shuai X, Gao J, Wang G, Tao K (2019). First-line systemic therapy for advanced gastric cancer: a systematic review and network meta-analysis. Ther Adv Med Oncol.

[21] (2023). Study Quality Assessment Tools | NHLBI, NIH. https://www.nhlbi.nih.gov/health-topics/study-quality-assessment-tools..

[22] DerSimonian R, Laird N (1986). Meta-analysis in clinical trials. Control Clin Trials.

[23] Begg CB, Mazumdar M (1994). Operating characteristics of a rank correlation test for publication bias. Biometrics.

[24] Egger M, Davey Smith G, Schneider M, Minder C (1997). Bias in meta-analysis detected by a simple, graphical test. BMJ.

[25] Maestro-Benedicto A, Vela P, de Frutos F (2022). Frequency of hereditary transthyretin amyloidosis among elderly patients with transthyretin cardiomyopathy. Eur J Heart Fail.

[26] Bishop E, Brown EE, Fajardo J, Barouch LA, Judge DP, Halushka MK (2018). Seven factors predict a delayed diagnosis of cardiac amyloidosis. Amyloid.

[27] Campagnolo M, Cacciavillani M, Cipriani A, Salvalaggio A, Castellani F, Pilichou K, Briani C (2023). Peripheral nerve involvement in wild-type transthyretin amyloidosis. Neurol Sci.

[28] Damy T, Costes B, Hagège AA (2016). Prevalence and clinical phenotype of hereditary transthyretin amyloid cardiomyopathy in patients with increased left ventricular wall thickness. Eur Heart J.

[29] Galat A, Guellich A, Bodez D (2016). Aortic stenosis and transthyretin cardiac amyloidosis: the chicken or the egg?. Eur Heart J.

[30] Ladefoged B, Clemmensen T, Dybro A (2023). Identification of wild-type transthyretin cardiac amyloidosis in patients with carpal tunnel syndrome surgery (CACTuS). ESC Heart Fail.

[31] Reyes BA, Ikram A, Sperry B, Shapiro DB, Hanna M, Seitz WH (2017). Carpal tunnel syndrome and amyloid cardiomyopathy. J Hand Surg Am.

[32] Shije JZ, Bautista MA, Smotherman C (2022). The frequency of V122I transthyretin mutation in a cohort of African American individuals with bilateral carpal tunnel syndrome. Front Neurol.

[33] Sperry BW, Reyes BA, Ikram A (2018). Tenosynovial and cardiac amyloidosis in patients undergoing carpal tunnel release. J Am Coll Cardiol.

[34] Sperry BW, Khedraki R, Gabrovsek A (2021). Cardiac amyloidosis screening at trigger finger release surgery. Am J Cardiol.

[35] Wang S, Peng W, Pang M (2022). Clinical profile and prognosis of hereditary transthyretin amyloid cardiomyopathy: a single-center study in South China. Front Cardiovasc Med.

[36] Itzhaki Ben Zadok O, Abelow A, Vaxman I (2020). Prior carpal tunnel syndrome and early concomitant echocardiographic findings among patients with cardiac amyloidosis. J Card Fail.

[37] Zegri-Reiriz I, de Haro-Del Moral FJ, Dominguez F (2019). Prevalence of cardiac amyloidosis in patients with carpal tunnel syndrome. J Cardiovasc Transl Res.

